# High Antimicrobial Consumption, Low Reported Infection Rates: An Ecological Analysis of HAI Surveillance Discordance Across EU/EEA Countries

**DOI:** 10.3390/antibiotics15070700

**Published:** 2026-07-17

**Authors:** Adriana Tatomirescu, Liviu Florian Tatomirescu, Suzana Turcu, Diana Ciuc

**Affiliations:** 1 ”C.F.2” Clinical Hospital, 011464 Bucharest, Romania; adrianamagdat@yahoo.com (A.T.); liviufloriant@yahoo.com (L.F.T.); diana_ciuc@yahoo.com (D.C.); 2 Faculty of Medicine, Carol Davila University of Medicine and Pharmacy, 050474 Bucharest, Romania; 3 ”Francisc Rainer” Institute of Anthropology, Romanian Academy, 050711 Bucharest, Romania; 4 Romanian Academy, School of Advanced Studies of the Romanian Academy, Doctoral School of Economic Sciences, National Institute for Economic Research “Costin C. Kirițescu”, “Victor Slăvescu” Centre for Financial and Monetary Research, 050711 Bucharest, Romania; 5 Faculty of Medicine, Titu Maiorescu University Bucharest, 031593 Bucharest, Romania

**Keywords:** healthcare-associated infections, antimicrobial consumption, HAI underreporting, surveillance underreporting, broad-spectrum antibiotics, Eastern Europe, outlier analysis, ESAC-Net, EU4Health

## Abstract

**Background/Objectives:** Healthcare-associated infections (HAI) affect an estimated 4.3 million patients annually in EU/EEA hospitals; yet, reported prevalence rates vary nearly fivefold across member states, a degree of variation inconsistent with clinical or epidemiological differences alone. Romania exemplifies this paradox: it records one of the lowest reported HAI prevalence rates in Europe while sustaining one of the highest antimicrobial consumption levels on the continent. This study examines whether antimicrobial consumption data can serve as a cross-check on reported HAI prevalence across EU/EEA countries and discusses implications for European funding mechanisms. **Methods:** A cross-sectional ecological analysis was conducted using data from 26 EU/EEA countries. HAI prevalence was extracted from the ECDC Point Prevalence Survey 2022–2023; antimicrobial consumption indicators from the ESAC-Net Annual Epidemiological Report 2023. Pearson and Spearman correlations, multiple linear regression with Cook’s distance diagnostics, bootstrap resampling, and k-means cluster analysis were performed. Variance inflation factors were computed to assess multicollinearity; community sector AMC was excluded from the final regression model due to near-perfect collinearity with total AMC. **Results:** No antimicrobial consumption indicator was significantly associated with reported HAI prevalence (total AMC: r = 0.274, *p* = 0.175; broad-spectrum hospital proportion: r = 0.082, *p* = 0.692); bootstrap confidence intervals confirmed the instability of all estimates. Romania and Bulgaria were identified as influential outliers whose consumption profiles are substantially inconsistent with their reported infection rates, with model-based estimates of 7.3% and 7.0% respectively against reported values of 3.1% and 3.7%. Cluster analysis placed both countries in an isolated group with the highest antimicrobial consumption and broad-spectrum hospital antibiotic use in the sample alongside the lowest reported HAI prevalence, a configuration not replicated elsewhere in the EU/EEA. **Conclusions**: The absence of a significant aggregate association between antimicrobial consumption and reported HAI prevalence, combined with the systematic identification of Romania and Bulgaria as influential observations whose consumption profiles are inconsistent with their reported infection rates, raises the hypothesis of surveillance underreporting as a plausible explanation, though the ecological design does not permit causal inference and alternative explanations, including differences in antimicrobial stewardship maturity and prescribing culture, cannot be excluded. Antimicrobial consumption indicators, particularly the proportion of broad-spectrum antibiotics in hospital use, may serve as a proxy for HAI burden where direct surveillance is incomplete, and their integration into European surveillance frameworks is warranted. These findings have direct policy relevance for the allocation of resources under EU4Health, EU-JAMRAI 2, and national recovery and resilience programmes, and support investment in surveillance reform as a precondition for effective infection prevention programmes.

## 1. Introduction

Healthcare-associated infections (HAI) affect an estimated 3.5 million patients annually in the European Union and European Economic Area (EU/EEA), contributing to approximately 90,000 deaths per year and generating direct healthcare costs in excess of EUR 1.5 billion [1,2]. Despite this burden, the quality and completeness of HAI surveillance data vary substantially across member states, complicating cross-national comparisons and hampering the development of evidence-based policy [3].

The European Centre for Disease Prevention and Control (ECDC) coordinates HAI surveillance primarily through point prevalence surveys (PPS), the most recent of which was conducted in 2022–2023 across 28 EU/EEA countries and estimated that HAI affected over 4.3 million patients annually in EU/EEA hospitals [4] a figure higher than earlier estimates reflecting both methodological refinements and expanded surveillance coverage. While this methodology provides a standardised framework, reported prevalence rates range from approximately 3% to nearly 14% across participating countries, a variation that cannot be explained by clinical or epidemiological factors alone [4]. Several countries in Eastern and South-Eastern Europe consistently report prevalence rates at the lower end of this distribution, in apparent contradiction to well-documented structural deficits in their healthcare systems, including insufficient staffing, suboptimal hygiene infrastructure, and high rates of antimicrobial resistance (AMR) [3,5,6,7]. Romania, for example, reported a HAI prevalence of 3.1% in the 2022–2023 PPS, yet simultaneously recorded one of the highest total antibiotic consumption rates in the EU (27.4 DDD per 1000 inhabitants per day) and a proportion of broad-spectrum antibiotics in hospitals of 60.1%, compared with the EU/EEA mean of 40.1% [8,9] versus a sample mean of 36.58% across the 26 countries included in this analysis. This discordance is difficult to reconcile with a genuinely low HAI burden and raises the hypothesis of systematic underreporting as a plausible explanation, though this cannot be established on the basis of ecological data alone.

Antimicrobial consumption, monitored systematically through the European Surveillance of Antimicrobial Consumption Network (ESAC-Net) and reported annually by the ECDC, offers a theoretically attractive surrogate indicator for HAI burden. Antibiotic use in the hospital sector is closely linked to the management of infections, including HAI, and broad-spectrum antibiotic consumption in particular reflects the empirical treatment of complex, often resistant, hospital-acquired pathogens [10,11]. Unlike HAI surveillance, which depends heavily on local reporting capacity and culture, AMC data are collected through standardised reimbursement and sales-based systems that are less susceptible to ascertainment bias [8].

The policy relevance of this question is considerable. The EU has committed substantial resources to combating HAI and AMR through the EU4Health Programme (EUR 4.4 billion, 2021–2027), EU-JAMRAI 2, and national recovery instruments such as the Romanian NRRP (Component 12, I2.4, EUR 150.38 million), as well as the Antimicrobial Resistance Council Recommendation adopted in 2023 [12,13,14]. However, the effectiveness of these investments depends on the availability of accurate baseline data. If HAI surveillance systematically underestimates the true burden in certain member states, resources may be misallocated and the impact of funded interventions will be difficult to evaluate [1,7,15].

If a country consumes among the highest volumes of broad-spectrum antibiotics in Europe yet reports among the lowest rates of healthcare-associated infections, one of two conclusions follows: either its hospitals are exceptionally effective at preventing infections despite treating the most resistant organisms, or its surveillance system is not capturing what is actually occurring. This study examines whether antimicrobial consumption indicators can serve as a cross-check on reported HAI prevalence across EU/EEA countries, identifies member states whose consumption profiles are inconsistent with their reported infection rates, and discusses the implications for European funding mechanisms targeting surveillance reform and infection prevention. Unlike prior studies, which have documented surveillance underreporting across EU/EEA member states primarily through qualitative evidence and expert opinion, the present approach offers a quantitative, replicable framework based exclusively on publicly available, standardised ECDC data.

## 2. Results

### 2.1. Descriptive Statistics

The analytical sample comprised 26 EU/EEA countries with complete data for both HAI prevalence and antimicrobial consumption indicators (Table 1).

Reported HAI prevalence ranged from 3.0% (Latvia) to 12.2% (Greece), with an unweighted mean of 6.62% (SD = 2.35). Total antimicrobial consumption varied nearly threefold across the sample, from 9.6 DDD per 1000 inhabitants per day in the Netherlands to 28.5 in Greece (mean 19.11, SD = 5.21). The proportion of broad-spectrum antibiotics in hospital sector consumption ranged from 14.2% (Czechia) to 65.0% (Bulgaria), with a mean of 36.58% (SD = 12.78). Romania recorded a total AMC of 27.4 DDD per 1000 inhabitants per day and a broad-spectrum hospital proportion of 60.1%, the second highest values in the sample for both indicators, alongside a reported HAI prevalence of 3.1%, among the lowest observed.

These findings are particularly relevant in the context of ongoing European investment in infection prevention infrastructure, as Romania and Bulgaria are both active participants in EU-JAMRAI 2 and beneficiaries of PNRR-funded healthcare investments during the period covered by this analysis.

### 2.2. Correlation Analysis

Pearson and Spearman correlation coefficients between reported HAI prevalence and each antimicrobial consumption indicator are presented in Table 2. None of the four AMC variables were significantly associated with HAI prevalence. Total AMC yielded a Pearson r of 0.274 (*p* = 0.175; Spearman rho = 0.240, *p* = 0.237), and community sector AMC produced nearly identical estimates (r = 0.273, *p* = 0.177), reflecting the near-perfect collinearity between the two measures (r = 1.00, *p* < 0.001), which precluded their simultaneous inclusion in subsequent regression models. Hospital sector AMC and the proportion of broad-spectrum antibiotics showed weaker and non-significant associations (r = 0.089, *p* = 0.667 and r = 0.082, *p* = 0.692, respectively).

**Figure 1:**
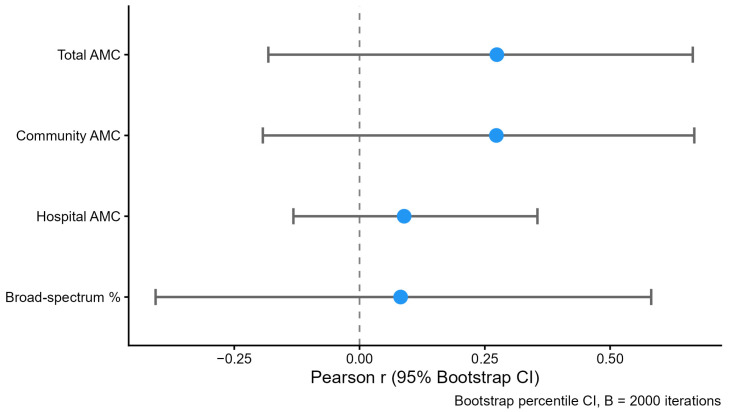
Bootstrap percentile confidence intervals for Pearson correlations between HAI prevalence and antimicrobial consumption indicators. Note: Vertical dashed line: r = 0. Bootstrap percentile method, B = 2000 iterations, seed = 42. Horizontal bars: 95% confidence intervals. AMC: antimicrobial consumption.

Given the modest sample size of 26 countries, the stability of correlation estimates was assessed through bootstrap resampling over 2000 iterations. All four 95% percentile confidence intervals crossed zero, confirming the absence of statistically reliable associations between AMC indicators and reported HAI prevalence (Table 3, Figure 1). The widest interval was observed for the broad-spectrum percentage [−0.407; 0.582], reflecting the disproportionate influence of Romania and Bulgaria on this estimator. For total and community AMC, the intervals were asymmetrically shifted toward positive values [−0.182; 0.665] and [−0.193; 0.668], suggesting a weak positive tendency that the current sample size is insufficient to establish with confidence.

### 2.3. Regression Analysis (Full Model)

The multiple linear regression model including total AMC and broad-spectrum hospital antibiotic proportion as predictors explained only 8.9% of the variance in reported HAI prevalence (R^2^ = 0.089, adjusted R^2^ = 0.010, F(2, 23) = 1.128, *p* = 0.341) (Table 4).

**Figure 2:**
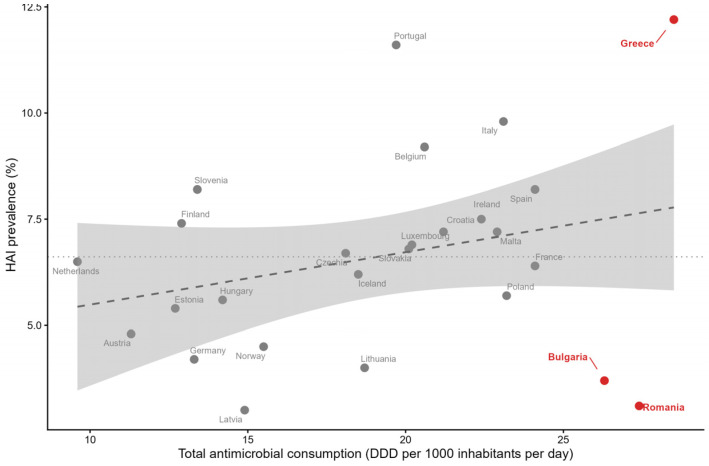
Total antimicrobial consumption versus reported HAI prevalence across 26 EU/EEA countries. Note: Dashed line: OLS regression fit (R^2^ = 0.089, *p* = 0.341). Shaded area: 95% confidence interval. Dotted horizontal line: unweighted sample mean HAI prevalence (6.62%). Red points: countries with Cook’s distance > 0.174 (Romania, Bulgaria, Greece). Sources: ECDC PPS 2022–2023; ESAC-Net 2023.

No individual predictor reached statistical significance: total AMC (β = 0.167, SE = 0.116, *p* = 0.162) and broad-spectrum percentage (β = −0.028, SE = 0.047, *p* = 0.558). The scatter plot of total AMC against HAI prevalence illustrates the weak and heterogeneous nature of this association across the full sample (Figure 2).

**Figure 3:**
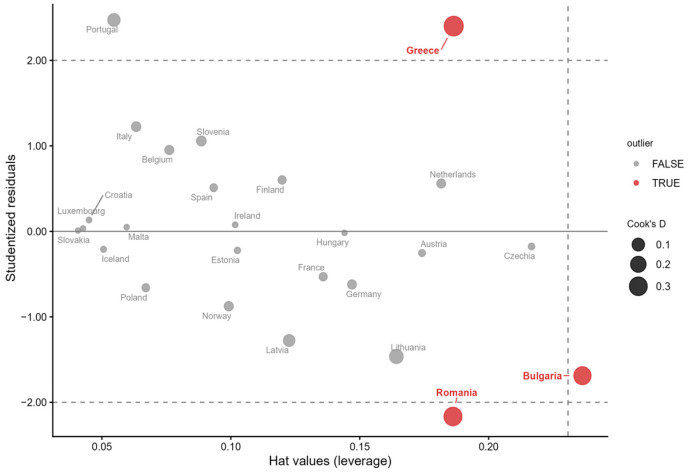
Influence plot: leverage, studentized residuals, and Cook’s distance for the full regression model. Note: Point size proportional to Cook’s distance. Red points: Cook’s D > 4/(n − k − 1) = 0.174. Dashed horizontal lines: | studentized residual | = 2. Dashed vertical line: leverage threshold = 2 (k + 1)/n. Model: HAI prevalence ~ total AMC + broad-spectrum hospital antibiotic proportion (n = 26).

Outlier diagnostics identified three countries with Cook’s distance exceeding the conventional threshold of 4/(n − k − 1) = 0.174. Romania (Cook’s D = 0.308) and Bulgaria (Cook’s D = 0.272) presented HAI prevalence substantially below model-based exploratory estimates (MBEEs) (MBEE of 7.3% versus reported 3.1% and MBEE of 7.0% versus reported 3.7%, respectively) yielding large negative studentized residuals (−2.01 and −1.63). Greece showed an opposite pattern, with a reported prevalence of 12.2% against a MBEE 7.6% (Cook’s D = 0.365, studentized residual +2.19). The leverage plot confirms that Romania and Bulgaria combine moderate-to-high leverage with large negative residuals, while Greece exerts influence through an extreme positive residual (Figure 3).

### 2.4. Sensitivity Analysis

When Romania, Bulgaria, and Greece were excluded, the proportion of variance explained by the model increased from 8.9% to 19.0% (adjusted R^2^ = 0.109, F(2, 20) = 2.352, *p* = 0.121), with total AMC approaching but not reaching conventional significance (β = 0.166, SE = 0.096, *p* = 0.100). The direction and magnitude of regression coefficients were largely unchanged, confirming that the overall model structure is not an artefact of these three observations. A quasi-Poisson model fitted to the full dataset yielded a pseudo-R^2^ of 0.093, consistent with the OLS estimates, and no predictor reached significance; the linear specification was therefore retained as the primary analytical framework.

Greece warrants separate consideration. Its reported prevalence of 12.2% substantially exceeds the model-based estimate of 7.6%, producing a large positive residual of opposite sign to those observed for Romania and Bulgaria. The underlying mechanism is therefore conceptually distinct: potential explanations include more intensive case ascertainment, differences in diagnostic culture, or a genuinely higher infection burden driven by patient case mix and healthcare system characteristics. Greece is excluded from the sensitivity model on statistical grounds alone and this exclusion carries no implication of a surveillance deficit analogous to that hypothesised for the two negative outliers.

### 2.5. Regional Analysis

**Figure 4:**
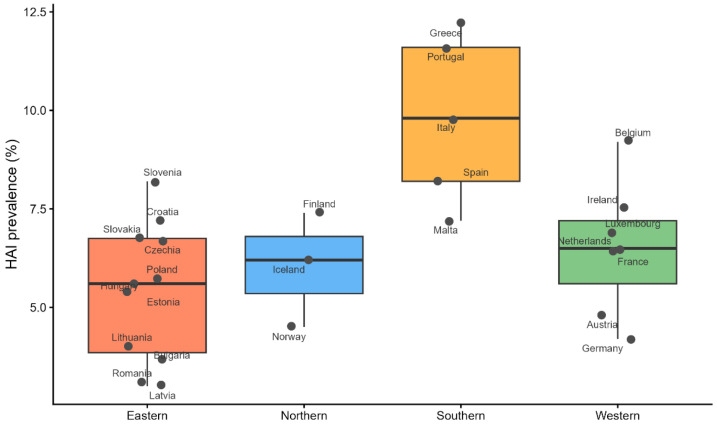
Distribution of reported HAI prevalence by geographic region. Note: Each point represents one country. Box: interquartile range; horizontal line: median; whiskers: 1.5 × IQR. Sources: ECDC PPS 2022–2023; ESAC-Net 2023.

Regional stratification revealed meaningful variation in HAI prevalence and AMC profiles across EU/EEA geographic areas (Table 5, Figure 4).

Southern European countries reported the highest mean HAI prevalence (9.80% ± 2.14) alongside elevated total AMC (23.66 ± 3.17 DDD/1000/day) and broad-spectrum use (47.9% ± 6.45%), a profile internally consistent with high infection burden and high antimicrobial use. Northern European countries presented the lowest mean AMC (15.63 ± 2.80) and broad-spectrum proportion (23.47% ± 5.98%), with moderate HAI rates (6.03% ± 1.46). Eastern European countries showed the greatest within-region heterogeneity (HAI SD = 1.75, BroadSpec% SD = 15.72), driven in part by the extreme profiles of Romania and Bulgaria, which are positioned at the opposite end of the HAI distribution from countries such as Slovenia (8.2%) and Croatia (7.2%) despite comparable or higher antimicrobial consumption. One-way ANOVA indicated statistically significant regional differences in reported HAI prevalence (F(3, 22) = 7.13, *p* = 0.002).

Post hoc Tukey testing identified Southern Europe as differing significantly from both Eastern (mean difference 4.40 percentage points, 95% CI [1.73; 7.07], *p* < 0.001) and Northern regions (mean difference 3.77 percentage points, 95% CI [0.15; 7.38], *p* = 0.039), as well as from Western Europe (mean difference 3.30 percentage points, 95% CI [0.40; 6.20], *p* = 0.022). Post hoc comparisons involving the Northern region should be interpreted with caution given the small group size (n = 3: Finland, Iceland, Norway), which limits the statistical power of pairwise tests. No significant differences were observed between Eastern, Northern, and Western regions.

### 2.6. Cluster Analysis

**Figure 5:**
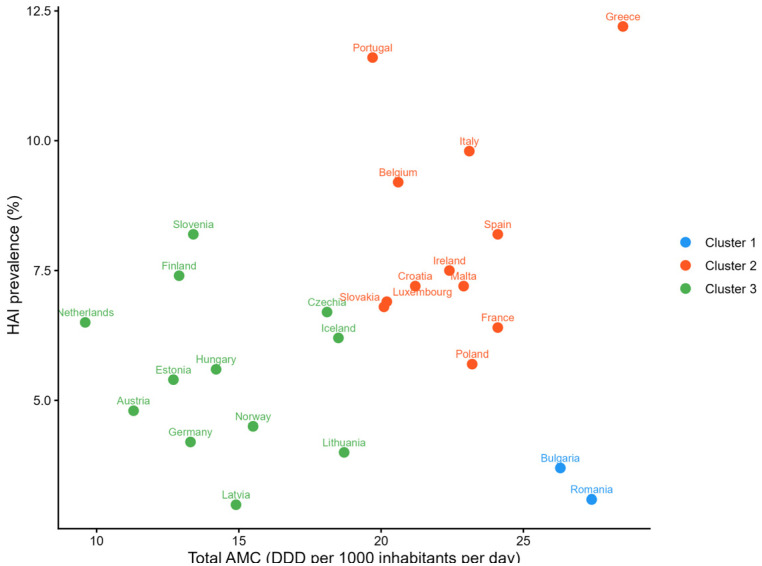
K-means cluster assignment of 26 EU/EEA countries based on HAI prevalence and antimicrobial consumption profile. Note: Cluster 1 (blue): Romania and Bulgaria. Cluster 2 (orange): high-AMC, high-HAI countries. Cluster 3 (green): low-AMC, moderate-HAI countries. Clustering based on standardised values; k = 3 determined by elbow criterion. Sources: ECDC PPS 2022–2023; ESAC-Net 2023.

K-means cluster analysis, with the optimal number of clusters determined as k = 3 by the elbow criterion, identified three distinct country profiles (Table 6, Figure 5). The quality of the three-cluster solution was assessed using the average silhouette width, which was 0.41, indicating a reasonable cluster structure. Cluster 1 (Romania and Bulgaria) showed a substantially higher silhouette width (0.83) than Clusters 2 and 3 (0.38 and 0.37, respectively), confirming that these two countries form a genuinely distinct and internally cohesive group relative to the rest of the sample.

Cluster 3 (n = 12) comprised countries with lower total AMC (mean 14.43 ± 2.87 DDD/1000/day), a modest proportion of broad-spectrum hospital antibiotics (28.52% ± 9.61), and intermediate HAI prevalence (5.54% ± 1.52), including Austria, Finland, Germany, the Netherlands, and Norway. Cluster 2 (n = 12) grouped countries with higher AMC (mean 22.51 ± 2.46), elevated broad-spectrum use (40.32% ± 8.30), and correspondingly higher reported HAI rates (8.22% ± 2.06), including Belgium, France, Greece, Italy, Portugal, and Spain, a profile consistent with high consumption driving measurable infection burden. Cluster 1 comprised exclusively Romania and Bulgaria, characterised by the highest total AMC (mean 26.85 ± 0.78 DDD/1000/day) and broad-spectrum proportion (62.55% ± 3.46) in the entire sample, yet the lowest reported HAI prevalence (3.40% ± 0.42). This configuration (extreme antimicrobial use co-occurring with minimal reported infection) is not replicated in any other country in the dataset and represents a statistically anomalous profile.

## 3. Discussion

This ecological analysis set out to examine whether antimicrobial consumption indicators could serve as a cross-check on reported HAI prevalence across EU/EEA countries. The regression model did not demonstrate a statistically significant association between AMC and reported HAI prevalence in the full sample, a finding that is itself substantively meaningful. The main finding of this study is not the association itself but the pattern of its absence: while a non-significant result can reflect limited statistical power or measurement error, the systematic identification of Romania and Bulgaria as influential outliers across multiple complementary analytical approaches points toward a structural rather than a random explanation for the discordance observed. This finding corroborates qualitative assessments of underreporting in Eastern European healthcare systems [5,6,7,16] and adds a quantitative, replicable dimension to a concern that has until now rested primarily on narrative evidence.

The ecological design of this study imposes important interpretive constraints. Country-level associations between antimicrobial consumption and reported HAI prevalence do not translate directly into mechanisms operating at the level of individual patients, wards or institutions—a limitation inherent to any aggregate analysis and not specific to the present dataset. The co-occurrence of high consumption and low reported prevalence at the national level cannot, on its own, serve as evidence of specific surveillance failures within individual hospitals, nor does it permit inferences about the prescribing decisions or infection control practices of particular clinicians or institutions. The data support a reproducible signal of aggregate discordance, confined to Romania and Bulgaria, that is unlikely to be attributable to random variation alone. Confirming whether this signal reflects genuine surveillance deficits will require investigation of disaggregated data, through hospital- and patient-level study designs. The association between broad-spectrum hospital antibiotic use and HAI prevalence, while not statistically significant in the full ecological model (r = 0.082, *p* = 0.692), merits careful interpretation rather than straightforward dismissal. The absence of a significant correlation across the full sample is itself informative: if AMC and HAI were moving in parallel across all countries, a meaningful association would be expected. The fact that it is not observed, despite a biologically plausible mechanism, points toward a structural distortion in the dependent variable rather than an absence of the underlying relationship. Broad-spectrum antibiotics are predominantly used for empirical treatment of severe, often hospital-acquired, infections caused by resistant organisms, precisely the pathogens that drive HAI incidence [10,11,17]. In countries where HAI surveillance is functioning adequately, this relationship should be detectable. The cluster and outlier analyses suggest that its absence at the aggregate level is driven by Romania and Bulgaria, whose extreme AMC profiles co-occur with implausibly low reported HAI rates, compressing the association toward the null. The broad-spectrum antibiotic proportion used as a predictor in this analysis corresponds conceptually to the Watch and Reserve categories of the WHO AWaRe classification, which has gained traction as a standardised indicator of prescribing appropriateness in European surveillance. Romania and Bulgaria recorded hospital broad-spectrum proportions of 60.1% and 65.0%, respectively, against an EU/EEA mean of 40.1%, a pattern dominated by Watch and Reserve agents and indicative of sustained selective pressure that is difficult to reconcile with the infection rates officially attributed to these settings, regardless of which explanatory hypothesis one finds more compelling.

High broad-spectrum antibiotic consumption in the hospital sector does not have a single explanatory pathway. Beyond infection incidence, consumption patterns are shaped by the maturity of local antimicrobial stewardship programmes, prescribing culture, the extent of empirical therapy, treatment duration practices, and access to rapid microbiological diagnostics [18,19,20,21]. In settings where stewardship infrastructure remains underdeveloped, these factors can sustain elevated consumption independently of the underlying infection burden. This possibility represents a legitimate competing explanation for the AMC profiles observed in Romania and Bulgaria, and one that the present ecological design cannot formally exclude. The absence of stewardship process indicators from the available datasets is accordingly acknowledged as a substantive limitation, and their incorporation into future cross-national surveillance analyses would materially improve the interpretability of consumption data as a proxy for HAI burden.

Romania’s antimicrobial consumption profile warrants particular attention in this context. With a total AMC of 27.4 DDD per 1000 inhabitants per day in 2023, representing a 6% increase from the 2019 baseline, Romania placed well above the EU mean of 20.0 as reported by ESAC-Net [7] (vs. a sample mean of 19.11 in the present dataset) and substantially further from the 2030 EU reduction target of 15.9 [8,9]. The proportion of broad-spectrum antibiotics in hospital sector consumption stood at 60.1%, the second highest value in the sample after Bulgaria and nearly 20 percentage points above the EU/EEA mean of 40.1%. In the regression model, Romania’s model-based exploratory estimate of HAI prevalence based on these AMC indicators was 7.3%, against a reported value of 3.1%, a discrepancy of 4.2 percentage points that generated a studentized residual of −2.01 and a Cook’s distance of 0.308, both exceeding conventional thresholds for influential observations. Given that the model explains only 8.9% of the variance in reported HAI prevalence and does not reach statistical significance, these estimates should be interpreted as exploratory benchmarks rather than precise predictions of expected infection burden. The k-means cluster analysis placed Romania alongside Bulgaria in an isolated cluster characterised by the highest AMC and broad-spectrum use in the entire sample, yet the lowest reported HAI prevalence, a configuration not replicated by any other EU/EEA country. These findings are difficult to reconcile with genuine differences in clinical practice, patient case mix, or infection control effectiveness alone, though such factors cannot be entirely excluded in an ecological analysis. A more plausible interpretation, consistent with previously documented constraints on HAI surveillance in Romania, points toward systematic underreporting: encompassing workforce capacity deficits, structural disincentives related to punitive sanctions, limited laboratory capacity and the absence of real-time electronic reporting systems [7,16]. This interpretation is corroborated by converging evidence from Romanian single-centre studies conducted across different hospital profiles and time periods. Duceac et al. reported an overall HAI incidence of 0.57% at a regional paediatric emergency hospital over a six-year period, substantially below both the national average and European benchmarks, explicitly attributing the discrepancy to dysfunctions in the clinical epidemiological monitoring and reporting system [22]. A longitudinal analysis of MDR-related HAIs in a Romanian tertiary-care hospital estimated a true HAI prevalence of 2.6% against an officially reported rate of 0.2–0.25%, a discrepancy the authors attribute to significant underreporting [23]. Luchian et al. documented Acinetobacter spp. HAIs with carbapenem-resistant MDR strains accounting for 88.27% of isolates in a Romanian neurological ICU [24], a resistance burden that, in the context of nationally reported HAI rates among the lowest in the EU/EEA, is difficult to reconcile without invoking surveillance gaps. The results of these studies suggest that the low HAI prevalence officially reported in Romania reflects, at least in part, incomplete ascertainment rather than a genuinely low infection burden.

### 3.1. Policy Implications and European Funding Opportunities

The identification of countries with probable HAI underreporting has direct implications for the allocation and design of European infection control programmes. Investment in surveillance reform is a necessary precondition for evaluating the effectiveness of other interventions, a point explicitly recognized in the 2023 Council Recommendation on antimicrobial resistance [13] and the ECDC’s PPS recommendations [4].

Several European funding instruments are directly relevant to addressing this surveillance gap. EU-JAMRAI 2, co-financed under the EU4Health Programme, supports 30 participating countries in updating national action plans on AMR and HAI, including mentorship and capacity-building for antimicrobial stewardship. Romania and Bulgaria (the two countries flagged as outliers in the present analysis) are both active participants [12]. The EU4Health Programme itself, with a total budget of EUR 4.4 billion for 2021–2027, allocates resources specifically to infection prevention and control practices and antimicrobial stewardship at all levels of care [12].

Within the funded programmes discussed above, clinical pharmacists represent a resource whose potential contribution to both stewardship and surveillance quality has been insufficiently acknowledged in policy frameworks. Their involvement in prescription audit, point prevalence survey coordination, and reporting validation has the potential to improve data completeness and stewardship adherence in comparable settings, and their formal integration into infection control structures should be considered a concrete and relatively low-cost component of any reform agenda targeting surveillance gaps in countries with identified reporting deficits.

Romania’s PNRR Component 12 (Health) allocated EUR 150.38 million under Investment I2.4 specifically for equipment and materials targeting the reduction in healthcare-associated infections in public hospitals, including the upgrading of microbiological laboratory capacity, with a minimum of 25 public healthcare facilities designated as beneficiaries [14]. More recently, national sources report that over 100 healthcare facilities have received upgraded microbiology equipment under the same programme, and a real-time HAI reporting system based on QR code technology has been piloted as part of the broader digital health investment stream. Comparable programme-level data for Bulgaria were not identified in publicly available sources at the time of writing this article.

These investments are well-targeted, but their success and the success of any future EU4Health calls will depend on robust outcome measurement. The present analysis suggests that AMC indicators, particularly broad-spectrum hospital use, could be systematically incorporated as secondary outcome measures in funded surveillance projects, providing a continuous, standardised cross-check against HAI reporting rates in countries with known surveillance limitations.

### 3.2. Limitations

Several limitations of this study must be acknowledged. First, the ecological design precludes causal inference; associations observed at the country level may not reflect individual-level relationships. Second, the sample size is constrained (n = 26), limiting statistical power and the number of covariates that can be included without overfitting. Third, HAI prevalence from a cross-sectional PPS may itself be subject to measurement error beyond systematic underreporting, including differences in case definitions, survey period, and hospital case mix across countries. Fourth, AMC data are subject to methodological heterogeneity in the classification of long-term care facility consumption across countries, as described in the ESAC-Net methodology [8]. Fifth, potential confounders (hospital bed density, healthcare expenditure as a proportion of GDP, and the proportion of elderly patients) were not included in the regression model, as these data were not available at the resolution and for the years corresponding to the primary data sources. Similarly, stewardship process indicators (programme maturity scores, adherence to formulary restrictions and audit-and-feedback implementation) were not available at the country level and represent a substantive limitation for the interpretation of AMC profiles as proxies for HAI burden. Future studies with access to these variables would benefit from more fully specified models. Finally, the absence of EARS-Net resistance data in the current analysis represents a gap that should be addressed in subsequent work, as AMR rates are likely to mediate part of the association between AMC and HAI burden [17,25]. This limitation is distinct from the hospital-level resistance profiles documented in Romanian single-centre studies, which are cited in the Discussion as contextual evidence rather than as model predictors.

## 4. Materials and Methods

### 4.1. Study Design

A cross-sectional ecological analysis was conducted at the country level. The unit of analysis was the individual EU/EEA member state. Countries were included if they had contributed data to both the ECDC PPS 2022–2023 and the ESAC-Net Annual Report 2023. Countries for which either HAI prevalence data or AMC data were unavailable (Cyprus, Sweden, Liechtenstein) were excluded, yielding an analytical sample of 26 countries.

### 4.2. Data Sources and Variables

HAI prevalence (%, 95% CI) was extracted from the country summary sheets of the ECDC Point Prevalence Survey of Healthcare-Associated Infections and Antimicrobial Use in European Acute Care Hospitals, 2022–2023 [4]. This variable constituted the dependent variable (Y) in all regression models.

Four AMC variables were obtained from the ESAC-Net Annual Epidemiological Report 2023 [8]: (1) total AMC (community and hospital combined), expressed as DDD per 1000 inhabitants per day (J01); (2) community sector AMC (DDD per 1000 inhabitants per day); (3) hospital sector AMC (DDD per 1000 inhabitants per day); and (4) the percentage of broad-spectrum antibiotics (glycopeptides, third- and fourth-generation cephalosporins, carbapenems, fluoroquinolones, polymyxins, piperacillin/enzyme inhibitor, linezolid, tedizolid, and daptomycin) out of total hospital sector antibiotic consumption. Variables (1)–(4) served as independent predictors (X1–X4). This grouping follows the classification adopted in ESAC-Net reporting conventions and is not arbitrary; the selected agents share the common characteristic of broad activity against resistant Gram-negative and Gram-positive pathogens typically encountered in hospital-acquired infections. A sensitivity analysis using carbapenem consumption as an alternative narrow indicator would be informative but was not feasible within the constraints of the publicly available dataset, which does not provide country-level disaggregation by individual antibiotic class for the hospital sector at the resolution required.

For imputed values (Cyprus 2023 = 2022 values; Sweden not available), only countries with observed data for both 2022 and 2023 were retained. Germany was excluded from hospital sector analyses due to missing historical data, consistent with ESAC-Net reporting conventions.

### 4.3. Statistical Analysis

Descriptive statistics were computed for all variables. The normality of residuals was assessed using the Shapiro–Wilk test. Pearson correlation coefficients were calculated between each AMC predictor and HAI prevalence; Spearman rank correlations were computed as robustness checks. A multiple linear regression model was fitted using ordinary least squares (OLS), with HAI prevalence as the dependent variable and total AMC, hospital AMC, and percentage of broad-spectrum antibiotics as predictors.

Variance inflation factors (VIF) were computed to assess multicollinearity When total AMC and community sector AMC were included simultaneously, both yielded VIF values far exceeding the threshold of 5 (VIF = 159.29 and 168.74, respectively), confirming near-perfect collinearity consistent with the observed correlation of r = 1.00 (*p* < 0.001) between the two variables; community sector AMC was therefore excluded from the final model. The retained predictors, total AMC and broad-spectrum hospital antibiotic proportion, showed negligible collinearity (VIF = 1.66 for both). Total AMC was preferred over hospital sector AMC as the primary consumption predictor given its stronger marginal correlation with reported HAI prevalence in this sample (r = 0.274 versus r = 0.089; Table 2). Standardised residuals were examined to assess model fit. Based on the existing literature [5,6,7,16,22,23,24], we hypothesised that certain Eastern European countries might exhibit inconsistency between their AMC profiles and reported HAI rates. Cook’s distance was used to identify influential observations empirically, without pre-selecting any country. All analyses were conducted in R version 4.3.2 (R Foundation for Statistical Computing, Vienna, Austria). Statistical significance was set at *p* < 0.05 (two-tailed).

### 4.4. Ethical Considerations

This study used exclusively publicly available, aggregated, country-level data. No individual patient data were used and no ethical approval was required.

## 5. Conclusions

This ecological analysis demonstrates that, at the aggregate level, antimicrobial consumption indicators do not show a statistically significant association with reported HAI prevalence across EU/EEA countries. Romania and Bulgaria emerge consistently across correlation analysis, regression diagnostics, and cluster analysis as countries whose consumption profiles are substantially inconsistent with their reported infection rates, a configuration not observed elsewhere in the sample. While alternative explanations cannot be excluded on the basis of ecological data alone, including differences in antimicrobial stewardship maturity, prescribing culture, diagnostic intensity, and microbiological testing rates, systematic underreporting of healthcare-associated infections remains the most plausible hypothesis consistent with the available evidence and one corroborated by converging findings from Romanian single-centre studies [22,23,24]. These findings support the integration of AMC-based indicators, particularly the proportion of broad-spectrum antibiotics in hospital use, into HAI surveillance frameworks as a complementary cross-check in settings where direct surveillance may be incomplete. They also underscore the need for sustained investment in surveillance reform as a precondition for evidence-based infection control policy in the EU. European funding mechanisms (including EU4Health, EU-JAMRAI 2, and national PNRR programmes) represent timely and well-targeted instruments for addressing this gap, provided that outcome measurement frameworks are strengthened commensurately and that reporting quality is treated as a primary objective rather than an assumed baseline.

## Figures and Tables

**Table 1:** Descriptive statistics for HAI prevalence and antimicrobial consumption indicators across 26 EU/EEA countries.

Variable	Mean	SD	Min	Max
HAI prevalence (%)	6.62	2.35	3.00	12.20
Total AMC (DDD/1000/day)	19.11	5.21	9.60	28.50
Community AMC (DDD/1000/day)	17.46	5.12	8.80	26.70
Hospital AMC (DDD/1000/day)	1.65	0.46	0.77	3.17
Broad-spectrum antibiotics (%)	36.58	12.78	14.20	65.00

Note: AMC: antimicrobial consumption; DDD: defined daily doses; SD: standard deviation. Data sources: ECDC Point Prevalence Survey of Healthcare-Associated Infections 2022–2023; ESAC-Net Annual Epidemiological Report 2023.

**Table 2:** Pearson and Spearman correlations between reported HAI prevalence and antimicrobial consumption indicators.

AMC Indicator	Pearson r	*p*-Value	Spearman rho	*p*-Value
Total AMC	0.274	0.1749	0.240	0.2370
Comm. AMC	0.273	0.1773	0.242	0.2336
Hosp. AMC	0.089	0.6672	0.203	0.3194
BroadSpec%	0.082	0.6921	0.068	0.7411

Note: *p*-values for Spearman correlations estimated using the asymptotic approximation in the presence of ties. HAI: healthcare-associated infections; AMC: antimicrobial consumption.

**Table 3:** Bootstrap percentile confidence intervals for Pearson correlations between HAI prevalence and antimicrobial consumption indicators.

AMC Indicator	Pearson r	95% Bootstrap CI (2000 Iterations)
Total AMC	0.274	[−0.182; 0.665]
Community AMC	0.273	[−0.193; 0.668]
Hospital AMC	0.089	[−0.132; 0.355]
Broad-spectrum %	0.082	[−0.407; 0.582]

Note: Bootstrap percentile method, B = 2000 iterations, seed = 42. All 95% confidence intervals include zero. AMC: antimicrobial consumption.

**Table 4:** Multiple linear regression results: full model and sensitivity analysis excluding outliers.

Parameter	Full Model (*n* = 26)	Model Without Outliers (*n* = 23)
Intercept	4.448 (SE = 1.805, 95% CI [0.713; 8.182])	2.859 (SE = 1.836, 95% CI [−0.970; 6.689])
AMC_total	β = 0.167, SE = 0.116, *p* = 0.162, 95% CI [−0.072; 0.406]	β = 0.166, SE = 0.096, *p* = 0.100, 95% CI [−0.035; 0.367]
BroadSpec_pct	β = −0.028, SE = 0.047, *p* = 0.558, 95% CI [−0.126; 0.070]	β = 0.024, SE = 0.045, *p* = 0.599, 95% CI [−0.070; 0.118]
R^2^	0.089	0.190
Adj. R^2^	0.010	0.109
F-statistic	1.128	2.352
*p*-value	0.341	0.121
n	26	23

Note: Outliers excluded from the sensitivity model: Romania, Bulgaria, Greece (Cook’s D > 0.174). SE: standard error. Quasi-Poisson model (pseudo-R^2^ = 0.093) yielded consistent results; the linear specification was retained as the primary model.

**Table 5:** HAI prevalence and antimicrobial consumption by geographic region.

Region	n	HAI Prev. Mean ± SD (%)	Total AMC Mean ± SD	BroadSpec% Mean ± SD
Eastern	11	5.4 ± 1.75	19.11 ± 5.09	37.39 ± 15.72
Northern	3	6.03 ± 1.46	15.63 ± 2.8	23.47 ± 5.98
Southern	5	9.8 ± 2.14	23.66 ± 3.17	47.9 ± 6.45
Western	7	6.5 ± 1.67	17.36 ± 5.81	32.86 ± 4.29

Note: Regional classification: Eastern (Bulgaria, Croatia, Czechia, Estonia, Hungary, Latvia, Lithuania, Poland, Romania, Slovakia, Slovenia); Northern (Finland, Iceland, Norway); Southern (Greece, Italy, Malta, Portugal, Spain); Western (Austria, Belgium, France, Germany, Ireland, Luxembourg, Netherlands).

**Table 6:** K-means cluster profiles based on HAI prevalence and antimicrobial consumption indicators.

Cluster	*n*	HAI Prev. Mean ± SD (%)	Total AMC Mean ± SD	Broad Spec% Mean ± SD	Countries
1	2	3.4 ± 0.42	26.85 ± 0.78	62.55 ± 3.46	Bulgaria, Romania
2	12	8.22 ± 2.06	22.51 ± 2.46	40.32 ± 8.3	Belgium, Croatia, France, Greece, Ireland, Italy, Luxembourg, Malta, Poland, Portugal, Slovakia, Spain
3	12	5.54 ± 1.52	14.43 ± 2.87	28.52 ± 9.61	Austria, Czechia, Estonia, Finland, Germany, Hungary, Iceland, Latvia, Lithuania, Netherlands, Norway, Slovenia

Note: Optimal number of clusters (k = 3) determined by the elbow criterion. Clustering performed on standardised values of HAI prevalence, total AMC, and broad-spectrum hospital antibiotic proportion. AMC: antimicrobial consumption; SD: standard deviation.

## Data Availability

All data used in this study are publicly available. HAI prevalence data are available from the ECDC PPS 2022–2023 country summary sheets at https://www.ecdc.europa.eu (accessed on 12 May 2026) AMC data are available from the ESAC-Net Annual Epidemiological Report 2023 at https://www.ecdc.europa.eu (accessed on 12 May 2026).

## Abbreviations

The following abbreviations are used in this manuscript:

AMC	Antimicrobial consumption
AMR	Antimicrobial resistance
AWaRe	Access, Watch and Reserve (WHO antibiotic classification)
DDD	Defined daily doses
ECDC	European Centre for Disease Prevention and Control
ESAC-Net	European Surveillance of Antimicrobial Consumption Network
EU	European Union
EEA	European Economic Area
EU-JAMRAI	Joint Action on AMR and Healthcare-Associated Infections
HAI	Healthcare-associated infections
IPC	Infection prevention and control
MBEE	Model-based exploratory estimate
PNRR	Planul National de Redresare si Rezilienta (National Recovery and Resilience Plan)
PPS	Point prevalence survey
OLS	Ordinary least squares
VIF	Variance inflation factor
